# Granuloma induced by sustained-release fluorouracil implants misdiagnosed as a hepatic tumor: A case report

**DOI:** 10.3892/ol.2014.2166

**Published:** 2014-05-22

**Authors:** DOU-SHENG BAI, SHENG-JIE JIN, RONG HE, GUO-QING JIANG, JIE YAO

**Affiliations:** Department of Hepatobiliary and Pancreatic Surgery, Northern Jiangsu People’s Hospital, Affiliated Hospital to Yangzhou University, Yangzhou, Jiangsu 225001, P.R. China

**Keywords:** fluorouracil, sustained-release implants, hepatic tumor, peritoneal interstitial chemotherapy

## Abstract

Sustained-release fluorouracil (FU) implants have been extensively used in peritoneal interstitial chemotherapy, and during surgery for gastrointestinal tumors, breast cancer and hepatic tumors. Currently, studies regarding the complications associated with sustained-release FU implants are rare. The present study describes the case of a 61-year-old male who presented with a space-occupying lesion of the left lobe of the liver six months after undergoing a radical total gastrectomy. Thus, laparoscopic exploration was performed to remove the tumor. Postoperative histological examination demonstrated that the lesion in the left lobe comprised of necrotic tissue with granulation tissue hyperplasia. Based on the surgical and postoperative histological findings, the mass was proposed to be due to a high concentration of local sustained-release FU implants. Furthermore, the drug was partially surrounded and had been insufficiently metabolized over a long time period, which was proposed to have caused necrosis, proliferation and fibrillation, and induced granuloma. In conclusion, local high concentrations of sustained-release FU implants may be associated with granuloma and this finding may enable improved management of sustained-release FU implants during surgery.

## Introduction

5-Fluorouracil (FU) is an antimetabolite agent, which is extensively used to treat gastrointestinal tumors and breast cancer. In the early 1990s, the concept of interstitial chemotherapy was proposed. Interstitial chemotherapy involves loading antineoplastic agents into degradable or non-degradable vehicles to provide a sustained drug release system to maintain a prolonged drug concentration at the site of implantation, as well as to reduce toxicity and achieve drug targeting (1). Since 2003, sustained-release FU implants have been extensively used in peritoneal interstitial chemotherapy, and during surgery for gastrointestinal tumors, breast cancer and hepatic tumors in China (2–4). The primary complications associated with peritoneal interstitial chemotherapy using sustained-release FU implants are chemical peritonitis, incision infection, anastomotic fistula and abdominal infection. To the best of our knowledge, this is the first report regarding sustained-release FU implants inducing granuloma. Patient provided written informed consent.

## Case report

A 61-year-old male presented with a space-occupying lesion of the left lobe of the liver, which was identified using computed tomography (CT) on July 27, 2013. The patient had undergone a total gastrectomy for an ulcerated adenocarcinoma of the greater curvature of the middle of the stomach six months previously at the Department of General Surgery at the First People’s Hospital of Yangzhou (Yangzhou, China). During the surgery, a 600-mg sustained-released FU implant (cylindrical granule, 4×0.8 mm) was implanted. A post-surgery histological examination revealed a moderately-low differentiated adenocarcinoma. No tenderness or palpable mass was detected in the abdomen of the patient. Upon admission to hospital, laboratory examinations revealed that the serum concentrations of carcinoembryonic antigen, α-feroprotein and carbohydrate antigen 19-9 were 1.6 ng/ml, 2.1 ng/ml and 3.2 IU/ml, respectively.

The patient underwent laparoscopic exploration in order to remove the liver tumor. During the surgery, a mass (diameter, 2.0×2.5 cm) was observed between the left lobe and the diaphragm, which presented with milky white necrotic matter and a cylindrical granule in the center. Ultrasonography indicated that no other tumors were present in the liver during the surgery.

A post-surgery histological examination demonstrated that the mass was granuloma, presenting with tissue necrosis at the center and granulation tissue hyperplasia, which was surrounded by hyperplastic fibrous tissue.

## Discussion

Locoregional recurrence and distant metastases are common following surgery for gastric adenocarcinoma. Thus, in the present case, the patient was initially diagnosed with liver metastases based on the history of gastric cancer and the findings of the CT scans. Radiofrequency ablation or alcohol injections are commonly used to treat single, small, metastatic liver lesions from gastric cancer. However, in the present case, the lesion was located on the surface of the left lateral lobe and there was adhesion to the intestine in the local area, thus, laparoscopic exploration rather than local treatment was used. The lesion was resected with minimal trauma.

**Figure 1:**
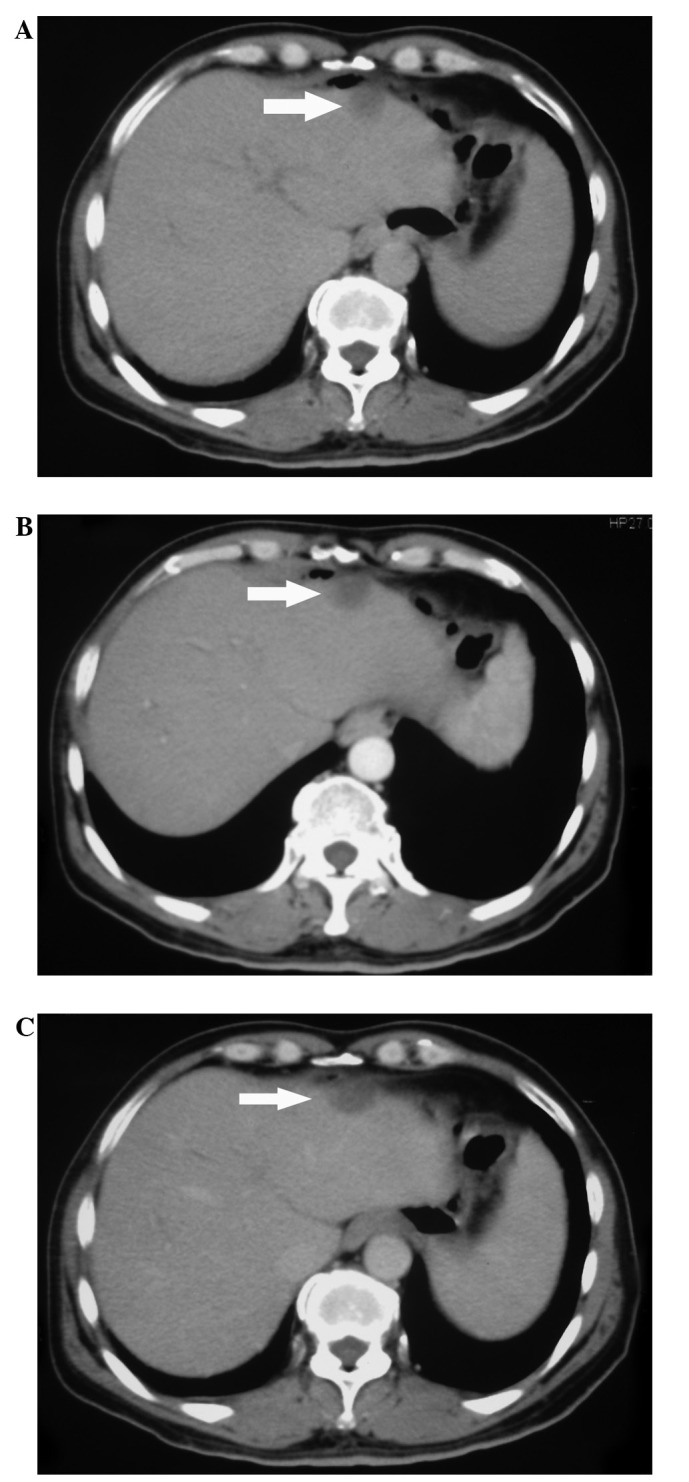
(A) Computed tomography (CT) scan showing the low-density liver lesion on the surface of the left lateral lobe and the adhesion to the intestine in the local area. (B and C) Contrast-enhanced CT scan demonstrating no marked change in intensification in the arterial and venous phases.

When gastrointestinal tumors metastasize to the liver, enhanced CT of the lesion edge commonly reveals intensification in the early period, however, in the later period, the density is low. In the present case, no obvious change in intensification was observed (Fig. 1). This may be be a difference in the representation of liver metastases and granuloma as observed by CT imaging. Furthermore, it demonstrates the difficulty in diagnosing liver metastases, particularly when the imaging results are not typical. Thus, treatment should not be administered prior to a pathological diagnosis.

A sustained-release method of 5-FU adminstration prevents the peak tissue levels that are induced by subconjunctival injections. Another potential advantage of this mode of administration is that 5-FU is delivered directly to the required site of action, further reducing the potential of toxic side-effects.

**Figure 2:**
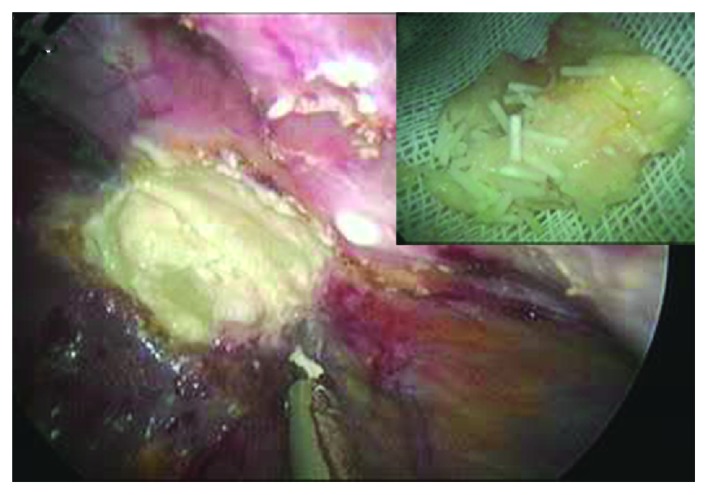
Lesion identified during laparoscopy. (Inset) The contents of the lesion.

In the present case, the cylindrical granule that was observed at the center of the granuloma was identified to be undegraded sustained-release FU implant polymers (Fig. 2). Thus, it was hypothesized that in the present case, the local concentration of implant polymers led to local tissue necrosis and the proliferation of granuloma and fibrous tissues. As a consequence, the proliferative tissue was surrounded and became granuloma, which was confirmed by post-surgery histological examination. The incidence of this type of complication is relatively low.

Biodegradable and non-biodegradable polymers are often used as implant base materials. The release pattern from biodegradable implants is controlled by the composition and molecular weight of the polymer, as well as the morphology, manufacturing technique and the structure of the implant. With developments in materials science, a novel biodegradable drug carrier, which is constructed using nano-controlled release technology, has been developed, which is capable of accessing the circulation of the human body and reaching the targeted area via vessels and cell absorption. This may be a promising targeting vector for use in the future (5–7).

In conclusion, the present study has shown a rare complication associated with sustained-release FU implants and has illustrated that higher local concentration implants may lead to granuloma. Therefore, the diagnosis of a metastatic tumor should be based on the results of pathological examination.
